# Parent-of-origin regulation by maternal auts2 shapes neurodevelopment and behavior in fish

**DOI:** 10.1186/s13059-025-03600-y

**Published:** 2025-05-09

**Authors:** Antoine Emile Clément, Constance Merdrignac, Sergi Roig Puiggros, Dorine Sévère, Aurélien Brionne, Thomas Lafond, Thaovi Nguyen, Jérôme Montfort, Cervin Guyomar, Alexandra Dauvé, Amaury Herpin, Denis Jabaudon, Violaine Colson, Florent Murat, Julien Bobe

**Affiliations:** 1 INRAE, LPGP UR1037, Fish Physiology and Genomics Institute, Rennes, France; 2 Department of Basic Neurosciences, University of Geneva, Geneva, Switzerland; 3 Sigenae, GenPhySE, INRAE, ENVT, Université de Toulouse, Toulouse, Castanet Tolosan France; 4 MGX-Montpellier GenomiX, University of Montpellier, CNRS, INSERM, Montpellier, France

**Keywords:** Non-genetic inheritance, Medaka, Intergenerational effect, Neurodevelopmental disorder, ASD

## Abstract

**Background:**

Parental experience can influence progeny behavior through gamete-mediated non-genetic inheritance, that is, mechanisms that do not involve changes in inherited DNA sequence. However, underlying mechanisms remain poorly understood in vertebrates, especially for maternal effects. Here, we use the medaka, a model fish species, to investigate the role of *auts2a*, the ortholog of human *AUTS2*, a gene repressed in the fish oocyte following maternal stress and associated with neurodevelopmental disorders.

**Results:**

We show that *auts2a* expression in the oocyte influences long-term progeny behavior, including anxiety-like behavior and environment recognition capabilities. Using single-nuclei RNA-sequencing, we reveal that maternal *auts2a* influences gene expression in neural cell populations during neurodevelopment. We also show that maternal *auts2a* knock-out triggers differences in maternally inherited factors, including early embryonic transcriptional and post-transcriptional regulators.

**Conclusions:**

Together, our results reveal the unsuspected role of an autism-related gene expressed in the mother’s oocyte in shaping progeny neurodevelopment and behavior. Finally, we report that *auts2a/AUTS2* is part of a group of evolutionarily conserved genes associated with human neurodevelopmental disorders and expressed in oocytes across species, from fish to mammals. These findings raise important questions about their potential role in the non-genetic regulation of progeny neurodevelopment and behavior in vertebrates.

**Supplementary Information:**

The online version contains supplementary material available at 10.1186/s13059-025-03600-y.

## Background

How environmental factors can intergenerationally shape progeny behavior is a long-standing question in animal biology that has been independently raised in various scientific fields. The impact of pre-fertilization events on the next generation has been documented in model organisms and humans [1, 2]. Ever since the early discovery that maternal stress can influence progeny behavior in rodents [3], the interest for the intergenerational impact of parental stress, experience, or exposure to environmental factors has progressively grown, including in humans. Yet, besides the acknowledgment that these intergenerational effects rely on non-genetic mechanisms (i.e., mechanisms that do not involve changes in inherited DNA sequence), our current understanding of these mechanisms remains extremely limited [2, 4]. Information that is non-genetically transmitted across generations is associated with parental experience and exposure to certain environments, but also with non-genetic consequences of parental mutations and polymorphism, because they can change the parental “intrinsic” environment [5, 6]. In mammals, decoding the mechanisms governing long-term maternal effects, such as behavior, is methodologically challenging due to factors like embryo implantation. For this reason, non-genetic inheritance mechanisms were mainly studied in males [1, 4]. To date, studies in males conducted in response to paternal stress consisted mostly in “Omics” screens that have yielded correlative results including changes in epigenetic marks, or sperm non-coding RNA levels [1, 7]. In contrast, studies specifically demonstrating the effect of a single parental non-genetic feature (i.e., gene expression or epigenetic mark) in the progeny remain scarce.

Several aspects of teleost fish models, including high fecundity and external embryonic development, make them extremely valuable to analyze the importance of maternal effects on progeny neurodevelopment and behavior in vertebrates. In contrast to most existing studies in which the impact of a parental stressor was used to reveal possible underlying mechanisms [8–10], we used an evolutionary perspective to target a specific mediator of maternal effects relevant to various vertebrate models and humans. We used the medaka (*Oryzias latipes*), a teleost model species with a short generation time and genetically determined sex. We focused on *auts2a* (i.e., the ortholog of *AUTS2*), a gene maternally repressed in the fish oocyte following maternal stress [11] and associated with neurodevelopmental disorders (NDDs), including autism spectrum disorders (ASD).

*AUTS2* (Activator Of Transcription And Developmental Regulator) was originally identified in monozygotic twins experiencing ASD and previously called “Autism Susceptibility Candidate 2” [12]. *AUTS2* has subsequently been associated with a diversity of diseases and phenotypes, including intellectual disability, microcephaly, short stature, and developmental delay now known as the AUTS2 syndrome [13]. During neurodevelopment, *AUTS2* regulates central nervous system gene expression through the PRC1.3/1.5-AUTS2 transcriptional regulatory complex [14–16]. *AUTS2* is involved in neural progenitor cell proliferation [17] and neuronal migration, as well as in axon and neurite elongation during neuronal differentiation [18]. The *Auts2* gene has been extremely well conserved during vertebrate evolution both in terms of genomic structure and protein domains [19, 20]. Existing data in various vertebrate models, including rodents and teleosts, have revealed conserved expression of *Auts2* during neurodevelopment [18, 21, 22]. Similarly, behavioral studies in various knock-out mutants have clearly established a link between *Auts2* and behavior [23–25], suggesting a major role of this gene in controlling vertebrate neurodevelopment and behavior consistent with our current understanding of the AUTS2 syndrome. However, existing studies in vertebrate models and humans have never considered the possibility that *Auts2* could be acting not only as a zygotically expressed regulator of neurodevelopment but also as an intergenerational driver of progeny neurodevelopment and behavior.

We established a unique model in which maternal or paternal *auts2a* was not functional (in the mother or the father specifically) but was functional in the progeny, to thoroughly characterize parental *auts2a* contribution to macroscopic traits, neurodevelopment, and long-term behavior in progeny. In our model, a copy of the gene (i.e., originating from one parent) was fully functional while the other copy (i.e., originating from the other parent) was not, which typically corresponds to the genetic situation (i.e., heterozygous mutations) of patients suffering from the AUTS2 syndrome [26, 27]. We generated a mutant line, in which 90% of *auts2a* genomic sequence was deleted. We show that maternal, but not paternal, *auts2a* loss-of-function (LOF) has a major impact on progeny neurodevelopment and behavior. In particular, our single-nuclei RNA-seq analysis reveals that maternal *auts2a* expression modulates gene expression across all neural cell populations during early neurodevelopment. Following maternal *auts2a* LOF, dysregulated genes associated with axon guidance, Wnt signaling, pathways regulating stem cell pluripotency, and neurodevelopmental disorders are overrepresented, including in neural cells during early neurodevelopment. We provide evidence indicating that *auts2a* expression in the mother (i.e., maternal *auts2a*) regulates the abundance of maternally inherited factors in the oocyte that control, in turn, early embryonic gene expression through transcriptional and post-transcriptional regulation. Finally, we show that *AUTS2* belongs to a group of 45 evolutionarily conserved maternally expressed oocyte genes that are associated with behavior and neurodevelopment in vertebrates and to a diversity of NDDs. We provide here a unique example of a non-genetic inheritance mechanism shaping long-term progeny behavior, through the modulation of gene expression in neural cells during early neurodevelopment. Together, these results shed new light on the non-genetic control of neurodevelopment and behavior in vertebrates.

## Results

### Biological model of parental auts2a expression

**Fig. 1:**
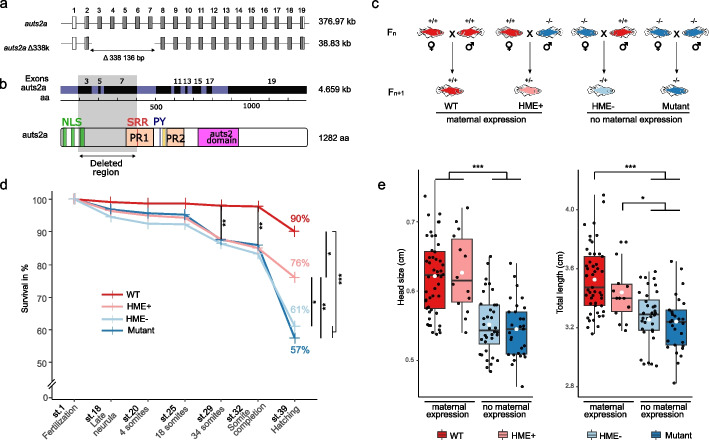
auts2a mutant line description and macroscopic phenotyping. **a** Scale-free diagram of genomic DNA of WT and mutant auts2a alleles in medaka. Shaded exons represent the translated parts of the gene. **b** Scaled diagram of the full-length WT and mutant predicted transcripts and proteins. Auts2a protein domains are represented by different colors. Nuclear Localization Signal (NLS, green), Serine Rich Repeat (SRR, red), Proline Rich region 1 and 2 (PR1 & PR2, orange), PY protein binding motif (PY, blue), Histidine repeats (HX, yellow), Auts2 family domain (auts2, pink). **c** Crossing scheme to generate individuals of various phenotypes with or without maternal auts2a expression. Deep red (wild-type, WT) and deep blue (mutants) individuals are homozygous. Light red (HME +) and blue (HME −) individuals are heterozygous. **d** Embryo survival throughout development. Stages of development according to Iwamatsu developmental table [31] are displayed on the X axis. Chi-square statistical test was performed between WT (n = 965), HME + (*n* = 681), HME − (*n* = 578), and mutant (*n* = 937) groups. **e** Head size and body length of 14-week-old fish. ANOVA and post hoc Tuckey’s tests were performed on WT (*n* = 49), HME + (*n* = 22), HME − (*n* = 36) and homozygous mutant (*n* = 29) groups. * *P* < 0.05, ** *P* < 0.01, *** *P* < 0.001.

To examine the role and importance of *auts2a* expression in the mother and in the father (i.e., maternal and paternal *auts2a*, respectively), we used the medaka (*Oryzias latipes*), a teleost model species in which a single ortholog of human *AUTS2* (named *auts2a*) is present [20]. Using CRISPR/Cas9-based genome editing, we generated the *auts2a* Δ338k mutant line. The *auts2a* Δ338k line harbors a deletion of 338,136 bp spanning exons 3–7 and large intronic regions (introns 2–7) (Fig. 1a and b, Additional file 1: Fig. S1a and b) associated with the AUTS2 syndrome in clinical studies [19, 28, 29]. This deletion would severely disrupt the canonical Auts2a protein but would not prevent the expression of shorter C-terminal isoforms previously described [18]. In homozygous mutants, *auts2a* expression could still be detected at both the mRNA and protein levels using mRNA probe and antibody directed against the C-terminal part of the gene, downstream of the mutation (Additional file 1: Fig. S1b and d). We did not observe any difference in mRNA or protein subcellular localization in mutant oocytes, compared to WT.

To specifically assess the role of maternal *auts2a* expression in the progeny, we crossed *auts2a* homozygous mutant (−/−) females with *auts2a* wild-type (WT, +/+) males to generate heterozygous (+/−) embryos lacking maternal *auts2a* expression (HME − group, maternal *auts2a* loss-of-function). We also performed the reciprocal crossing to generate heterozygous embryos (+/−) with maternal *auts2a* expression (HME + group, wild-type *auts2a* is expressed in the mother) (Fig. 1c). Comparing these heterozygous individuals lacking or not maternal *auts2a* expression allowed us to specifically assess the impact of maternal *auts2a* loss-of-function (LOF) in fish exhibiting the same genotype. This heterozygous state is highly relevant as it also corresponds to documented *AUTS2* mutations in human patients [13, 26, 27, 30]. In addition, we also used WT (+/+) and homozygous mutant (−/−) individuals in which both parents were either homozygous mutants (mutant group) or homozygous wild-type (WT group).

### Maternal control of macroscopic traits

In humans and other vertebrates, mutations in *Auts2* are associated with reduced survival [32], body length, and head size [13, 15, 33]. However, the contribution of maternal or paternal mutations to these phenotypes has never been assessed. To characterize the contribution of maternal *vs*. paternal *auts2a* LOF to developmental success and macroscopic traits, we monitored embryonic survival throughout development, as well as head and body size in adult progeny. No differences in embryonic survival were observed up to neural tube formation (i.e., stage 25) among the four experimental groups (WT, HME +, HME −, mutant). Differences in embryonic survival between groups emerged during the late embryonic brain formation (stage 29). At this stage, a lower embryonic survival (chi square, *P* = 5.7 e − 20) was observed in the three mutant groups (HME +, HME −, and mutant) in comparison to WT (Fig. 1d). This lower embryonic survival in the three mutant groups compared to WT remained significant until hatching (chi square, *P* = 1.6 e − 14, 5.7 e − 42, and 5.7 e − 42 for WT vs HME +, HME −, and mutant, respectively). At hatching, we observed further differences with a significantly lower survival in HME − and mutants in comparison to HME + (chi square, *P* = 9.4 e − 9 and 1.0 e − 14 for HME − and mutant, respectively). In contrast, no difference was observed between groups lacking maternal *auts2a* expression (i.e., mutant and HME − groups, chi square, *P* = 0.17). In adult individuals, we observed reduced head size (Tukey’s post hoc test, *P* < 1.0E − 7) and total length (Tukey’s post hoc test, *P* = 1.4 e − 6) in the mutant group compared to WT (Fig. 1e). A similar profile was observed between HME + and HME − (which have the same genotype) with a significantly smaller head size (Tukey’s post hoc test, *P* = 2.8 e − 5) and body length (Tukey’s post hoc test, *P* = 0.047) in the HME − group compared to HME +. In contrast, no differences in head size or body length were observed between groups with maternal *auts2a* expression (i.e., WT and HME +) or between groups lacking maternal *auts2a* expression (i.e., HME − and mutant), indicating that maternal *auts2a* expression drives these macroscopic phenotypes.

### Maternal control of progeny behavior

**Fig. 2:**
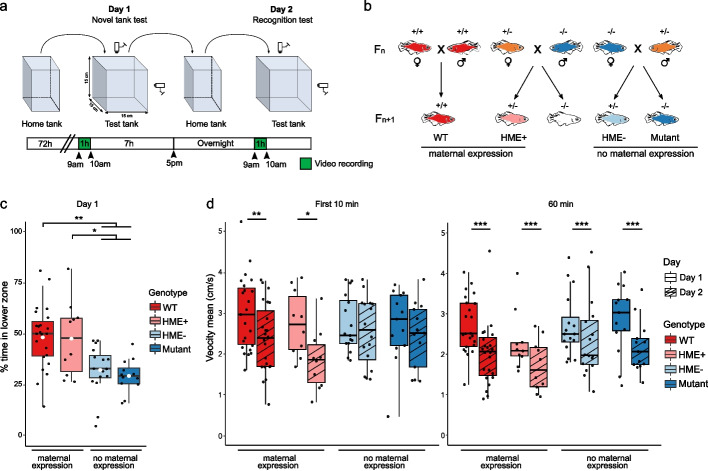
Long term behavioral phenotypes.** a** Schematic representation of behavioral tests. **b** Crossing of individuals used for behavioral test to assess the impact of maternal *auts2a* expression. Orange individuals are heterozygous and originate from heterozygous *auts2a* parents. **c** Percent of time spent in lower zone in the novel tank test and** d** mean velocity on day 1 and day 2, 10 or 60 min after the beginning of the experiment. ANOVA and post hoc Tukey’s statistical test were performed on WT (*n* = 23), HME + (*n* = 10), HME − (*n* = 16), and mutant (*n* = 13) groups. *P < 0.05, **P < 0.01, ***P < 0.001

To determine the contribution of maternal and paternal *auts2a* to long-term (i.e., adult) progeny behavior, we used a novel tank test to estimate anxiety-like behavior and a recognition test to evaluate environment recognition abilities (Fig. 2a). To minimize possible tank effects, the crossing scheme (Fig. 2b) was slightly modified (see Methods for details) in order to raise half of the WT individuals with fish of the HME + group and the other half of the WT individuals with fish of the HME − and homozygous mutant groups. To minimize experimental bias and ensure a fully blinded study design, fish were genotyped only after behavioral testing.

In the novel tank test, time spent in the lower half zone of the tank was used as an indicator of anxiety-like behavior [34]. We observed a significantly reduced time spent in the lower half zone in HME − and mutant groups in comparison to both WT (Tukey’s test, *P* = 3.5E − 3 and 1.4E − 3 for WT vs HME − and mutant, respectively) and HME + (Tukey’s test, *P* = 0.035 and 0.014 for HME + vs HME − and mutant, respectively) groups (Fig. 2c). In contrast, no difference in the time spent in the lower half zone was observed between groups with maternal *auts2a* expression (i.e., WT and HME +) or between groups without maternal *auts2a* expression (i.e., HME − and mutant). Together, these results indicate that the absence of maternal *auts2a* expression triggers reduced anxiety-like behavior in the progeny, regardless of the fish genotype.

To determine their ability to recognize their environment, fish were placed back in the test tank the following day (Fig. 2a). Their velocity mean was used as an estimator of their exploratory behavior, as velocity typically decreases after a while in a known environment [34]. On day 2, after 10 min in the test tank, fish of the WT and HME + groups, but not of the HME − and mutant groups, exhibited a reduced velocity in comparison to day 1 (Fig. 2d). After 60 min in the tank test, all experimental group exhibited a significantly reduced velocity in comparison to day 1 (Fig. 2d). Together, these results indicate that the absence of maternal *auts2a* expression impairs the progeny’s ability to recognize its environment, regardless of the fish genotype. These observations indicate that maternal *auts2a* expression is required for proper environmental recognition capabilities in the progeny.

To test whether these conclusions were also valid earlier in development, we performed a thigmotaxis (or “wall-hugging”) assay as previously described [35] using 4 days post-hatching larvae. This assay evaluates basic locomotion and uses the time spent along the walls of the well (i.e., in the thigmotaxis zone) as an indicator of anxiety-like behavior. We compared the response to a 10-min stressing dark challenge between HME + and HME − individuals (Additional file 2: Fig. S2). We found that HME − individuals spent more time in the inner zone of the well compared to their HME + conspecifics (Additional file 2: Fig. S2c, *t*-test, *P* = 0.007). These data show that the reduced anxiety-like behavior observed in adult fish lacking maternal *auts2a* expression is already detectable by 4 days post-hatching.

### Maternal auts2a expression regulates progeny neurodevelopment

#### Global impact during neurodevelopment

**Fig. 3:**
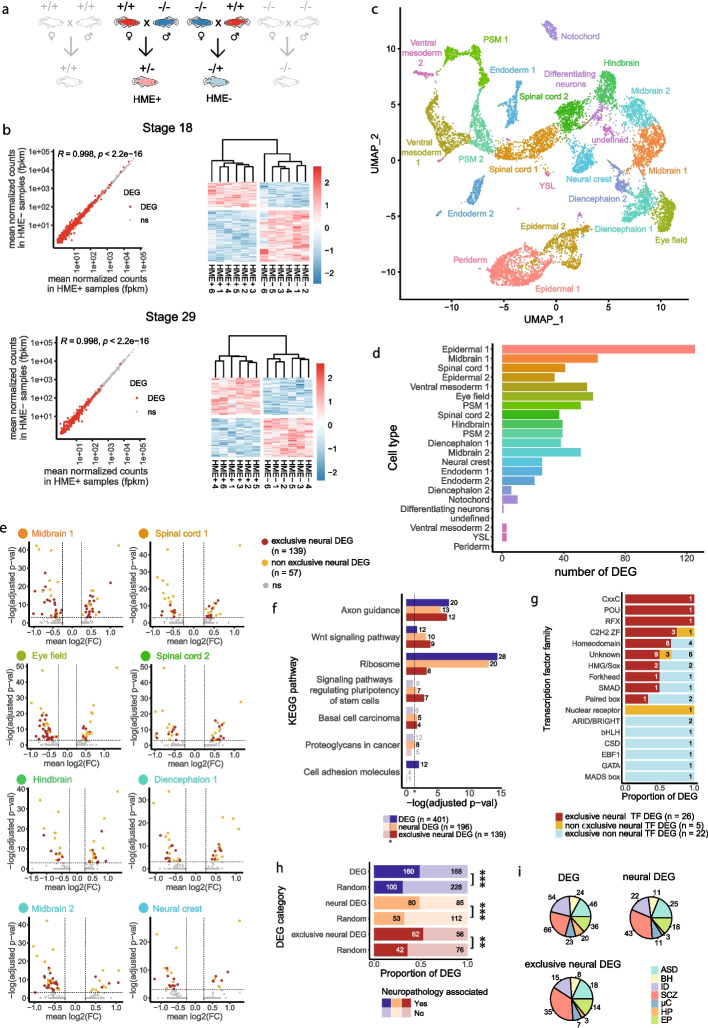
Transcriptomic analysis. **a** Crossing scheme to generate heterozygous individuals with (HME +) or lacking (HME −) maternal *auts2a* expression. **b** Bulk RNA-seq analysis of HME + and HME − progeny at early (stage 18, late neurula) and late (stage 29, late embryonic brain formation) stages of neurodevelopment. Mean normalized counts and Spearman correlation are displayed. Heatmaps display DEGs rlog scaled expression in HME − and HME + samples. **c** Single-nuclei RNA-seq clustering at late neurula stage (st 18). UMAP constructed in dimensionality-reduced PCA defined by highly variable genes from integrated HME + and HME − datasets. Cells sharing the same color have the same cellular identity determined from expressed marker genes (Additional file 1: Fig. S1a). **d** Single-nuclei DEGs between HME + and HME − embryos distribution. Bar plot displays number of single-nuclei DEGs for each cluster. Bar colors match with the colors of the UMAP clusters. Cell types are ordered from top to bottom by decreasing abundance. **e** Single-nuclei DEGs between HME + and HME − embryos at stage 18 in neural populations. Volcano plots display single-nuclei DEGs for neural clusters. HME + was used as the control condition. The colors of the cell type name in each sub-panel matches the colors of the UMAP clusters in panels **c** and **d**.** f** Dysregulated KEGG molecular signaling pathways. Bar plots display the enrichment of single-nuclei DEGs with identified human orthologs belonging to the KEGG signaling pathways database. The number of DEGs for each pathway is indicated. The dashed vertical line shows significance threshold. **g** Bar plots display the proportion of single-nuclei DEGs with identified human orthologs for each transcription factor family. The number of DEGs for each transcription factor family is indicated. Unknown transcription factors lack a canonical DNA binding domain. **h** Bar plots display the number of DEGs with identified human orthologs associated with NDDs for all single-nuclei DEGs (dark blue), neural DEGs (orange) and exclusive neural DEGs (red) group. NDDs include ASD, aura, behavior alterations, cognition disorders, delirium, developmental disorders, epilepsy, Huntington, hyperactivity, hyperopia, hypotonia, intellectual disability, language disabilities, mental retardation, microcephaly, nervous system disorders, neurodevelopment disorders, sleep apnea, and schizophrenia. Chi square analysis was performed against a random selection of genes in the genome (see Methods). *P < 0.05, **P < 0.01, *** *P* < 0.001. **i** Neuropathology associated with DEGs distribution. Pie charts display the distribution of neuropathology associated DEGs for all single-nuclei DEGs, neural DEGs and exclusive neural DEG group. Numbers correspond to the number of DEGs associated with the seven main neuropathologies for each DEG group. ASD: Autism Spectrum Disorders, BH: behavior, ID: intellectual disability, SCZ: schizophrenia, µC: microcephaly, HP: hypotonia, EP: epilepsy

To determine the contribution of maternal *auts2a* expression to neurodevelopment, the embryo transcriptome was studied at the late neurula stage (i.e., stage 18, early stage of neurodevelopment) [31] (Additional file 3: Table S1) and during late embryonic brain formation (i.e., stage 29, late stage of neurodevelopment) [31] (Additional file 4: Table S2). Bulk RNA-seq analysis was performed for the heterozygous groups (HME + and HME −) in which maternal *auts2a* expression, but not the progeny genotype, varied (Fig. 3a). This model corresponds to pathological cases in which only one copy of the human *AUTS2* gene is usually mutated [13, 26]. We observed a strong correlation in gene expression (Spearman correlation, R = 0.998, *p* < 2.2E − 16) between the HME + and HME − embryos at both early and late stages of neurodevelopment (Fig. 3b). When examining the expression of the 487 (stage 18, late neurula) and 277 (stage 29, late embryonic brain formation) differentially expressed genes (DEGs, with an adjusted *p*-value < 0.05), we observed a distinct clustering of HME + samples as a group and HME − samples forming a separate cluster. This clustering highlights an existing transcriptomic signature that clearly distinguishes HME + from HME − embryos at both early and late neurodevelopmental stages (Fig. 3b). The higher number of DEGs at the early stage (i.e., stage 18, late neurula), compared to the late stage of neurodevelopment (i.e., stage 29, late embryonic brain formation), suggests that the absence of maternal *auts2a* expression primarily influences gene expression at early stages of neurodevelopment (i.e., at stage 18, late neurula).

#### Impact on neural cell populations

To further explore these subtle, yet significant, differences, we performed single-nuclei RNA sequencing to investigate underlying variations at the cellular level. This analysis was conducted for HME + and HME − embryos at the early stage of neurodevelopment (i.e., stage 18, late neurula), given the higher number of DEGs at this stage in the bulk RNA-seq analysis. It is worth noting that no difference in *auts2a* expression was observed on the 338 kb deletion site among the HME +, HME −, and WT groups at that stage and that no expression was detected in mutant fish (Additional file 1: Fig. S1c). We successfully identified 20 distinct cell types from the main embryonic layers, including nine neural (diencephalon, midbrain, hindbrain, spinal cord, differentiating neurons, eye field, neural crest), three epidermal (epidermal, periderm), five mesodermal (notochord, ventral mesoderm, presomitic mesoderm), two endodermal (endoderm), and one yolk syncytial layer populations (Additional file 5: Fig. S3a, Additional file 6: Table S3). Cell populations are organized along the antero-posterior axis of the embryo, spanning from right to left of the UMAP. The anterior and mid neural clusters are positioned on the right, the posterior neural clusters on the center, and the mesodermal clusters are located on the left. Endodermal and epidermal clusters are distinct from this antero-posterior continuum (Fig. 3c). We identified DEGs between HME + and HME − cells for each cell type and obtained 401 DEGs distributed among all cell populations except for the periderm for which we did not identify any DEGs (Fig. 3d, Additional file 7: Table S4). More specifically, we identified 196 neural DEGs (i.e., with an adjusted *P*-value < 0.05 in at least one neural population) and 139 exclusively neural DEGs (i.e., with an adjusted *P*-value < 0.05 in at least one neural population and not differentially expressed in any non-neural population). No differences in *auts2*a expression levels were observed in neural populations, indicating that, in our heterozygous model, the expression level of *auts2a* does not depend on whether the allele is of maternal or paternal origin. No notable differences in cellular composition were observed between the HME + and HME − embryos, aligning with the subtle differences observed in the bulk transcriptomic analysis conducted on the entire embryo scale (Additional file 5: Fig. S3b-c). Moreover, when compared to bulk RNA sequencing analysis, differences in gene expression were exacerbated when examined at the cellular level, with high fold-changes between HME + and HME − embryos (Fig. 3e). Overall, these findings highlight that maternal *auts2a* LOF significantly impacts gene expression at the cellular level in the progeny, including in all neural cell populations during early neurodevelopment (i.e., at stage 18, late neurula stage). They also suggest that observed differences are not linked to any parent-of-origin bias in *auts2a* expression during neurodevelopment.

#### Dysregulated molecular signaling pathways during neurodevelopment

To further characterize dysregulated molecular processes in the absence of maternal *auts2a* expression, we investigated the enrichment of human orthologs of the identified single-nuclei DEGs in signaling pathways using the KEGG database (https://www.genome.jp/kegg/pathway.html). When comparing all single-nuclei DEGs between heterozygous individuals lacking (HME −) or not (HME +) maternal *auts2a* expression, we observed an enrichment for “ribosome,” “axon guidance,” “cell adhesion molecules,” and “Wnt signaling” pathways. Similar enrichment patterns emerged when examining neural DEGs (i.e., with an adjusted *P*-value < 0.05 in at least one neural population), displaying an even more pronounced enrichment (compared to all single-nuclei DEGs) in the Wnt signaling pathway and the appearance of a significant enrichment in the “signaling pathways regulating pluripotency of stem cells.” Exclusive neural DEGs (i.e., with an adjusted *P*-value < 0.05 in at least one neural population and not differentially expressed in any non-neural population) exhibited a more pronounced increase of the “Wnt signaling pathway” and “signaling pathways regulating pluripotency of stem cells” enrichment as well as a strong decrease of the “ribosome” pathway enrichment (compared to all single-nuclei DEGs and neural DEGs). The enrichment in the “axon guidance” pathway remained high (Fig. 3f, Additional file 8: Table S5).

Together, these findings reveal that genes that are regulated by maternal *auts2a* are enriched in pathways known to orchestrate intricate cell and tissue interactions governing development (such as Wnt), including neurodevelopment (axon guidance). This enrichment becomes even more prominent when focusing specifically on genes under maternal *auts2a* control in neural cells.

#### Dysregulated transcription factors during neurodevelopment

Because maternal *auts2a* expression was able to influence the expression of several hundreds of genes early during neurodevelopment (i.e., stage 18, late neurula), we specifically investigated transcription factors and monitored the orthologs of all inventoried human transcription factors [36]. When comparing all single-nuclei DEGs between heterozygous individuals lacking (HME −) or not (HME +) maternal *auts2a* expression, we observed a dysregulation of a large number of transcription factors (*n* = 53, 13.2% of all single-nuclei DEGs) including 26 (18.7% of exclusive neural DEG) exclusively differentially expressed in neural cells. Specifically, we noted that specific families of transcription factors exhibit differential expression exclusively in neural cells (i.e., CxxC, POU, RFX) or exclusively in non-neural cells (i.e., ARID/BRIGHT, bHLH, CSD, EBF1, GATA, MADS box). In contrast, transcription factors from other families were differentially expressed in neural as well as non-neural cells (i.e., C2H2 ZF, homeodomain, HMG/Sox, forkhead, SMAD, paired box, nuclear receptor, unknown DNA binding domain family) (Fig. 3g, Additional file 8: Table S5). Our analysis revealed that the lack of maternal *auts2a* expression triggers the dysregulation of several key transcription factor families in neural cells during neurodevelopment, in a cell type-dependent manner.

#### Dysregulated genes associated with NDDs

To explore links between maternal *auts2a* expression and molecular mechanisms linked to NDDs, we used the DisGeNET database that covers more than 24,000 diseases and traits, 17,000 genes and 117,000 genomic variants [37]. From the single-nuclei analysis, at the late neurula stage (i.e., stage 18, early neurodevelopment), a highly significant proportion (48.7%, *P* = 1,47E − 06) of the 328 single-nuclei DEGs human orthologs were associated with NDDs, in comparison to 30.5% for a random selection of 328 genes in the genome (Fig. 3h, Additional file 8: Table S5). This strong proportion of single-nuclei DEGs associated with NDDs was also observed when looking at the 185 neural DEGs human orthologs (48.5%, *P* = 2.19E − 6) and at the 118 exclusively neural DEGs human orthologs (52.5%, *P* = 0.0087) at the late neurula stage (Fig. 3h). Among the genes identified, some were associated with various pathologies including autism spectrum disorders (ASDs), behavioral abnormalities (BH), epilepsy (EP), hypotonia (HP), intellectual disability (ID), microcephaly (µC), and schizophrenia (SCZ) (Fig. 3i). Collectively, these findings highlight that maternal *auts2a* LOF dysregulates the expression of neural genes associated with NDDs during early neurodevelopment (i.e., late neurula).

**Fig. 4:**
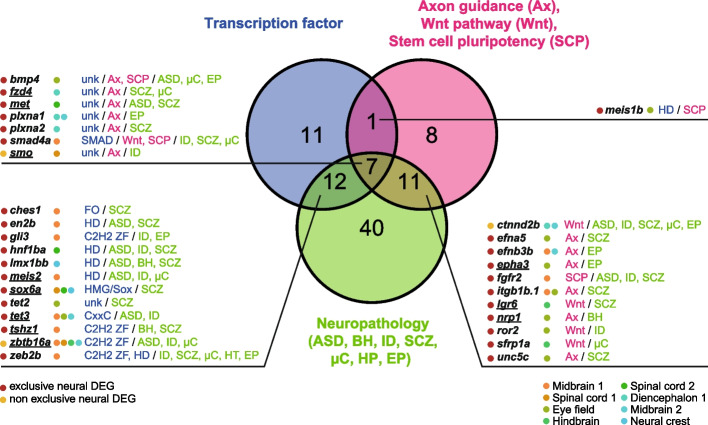
Neural DEGs. A Venn diagram displays the number of single-nuclei neural DEGs (i.e., with an adjusted *P*-value < 0.05 in at least one neural population) whose human orthologs have been identified as transcription factors (C2H2 ZF: C2H2 zinc finger, FO: forkhead, HD: homeodomain, HMG: High-Mobility Group, unk: unknown DNA binding domain), KEGG pathways (Ax: Axon guidance, Wnt: Wnt signaling pathway, SCP: signaling pathways regulating pluripotency of stem cells) and neuropathologies (ASD: autism spectrum disorders, BH: behavior, ID: intellectual delay, SCZ: schizophrenia, µC: microcephaly, HP: hypotonia, EP: epilepsy)

Together, our analyses highlighted neural DEGs of particular interest. Sets of these genes have been identified as transcription factors, are parts of enriched pathways of interest (i.e., axon guidance, Wnt signaling pathway, and/or regulating stem cell pluripotency), and/or have been associated with relevant NDDs (ASD, behavior, intellectual disability, schizophrenia, microcephaly, hypotonia, and/or epilepsy) (Fig. 4, Additional file 8: Table S5). We identified highly significant neural DEGs (with an adjusted *P*-value < 0.01 and an absolute fold change > 2 or an adjusted *P*-value < 0.001 and an absolute fold change > 1.5). Among others, noteworthy examples include a member of MET signaling (*met*) known to be involved in several neurodevelopment events including in early neuronal growth [38] and associated with ASD [39–42]. We could also highlight an EPH receptor (*epha3*) implicated in mediating developmental events, including in synaptic morphology, function, and plasticity [43, 44]. It is also worth noting a cell-surface receptor (*nrp1*) involved in the formation neuronal circuits [45] and transcription factors (*meis2*, *sox6a*) responsible for proper neurodevelopment [46–49] (Fig. 4, Additional file 8: Table S5).

### Non-genetic inheritance mechanisms

**Fig. 5:**
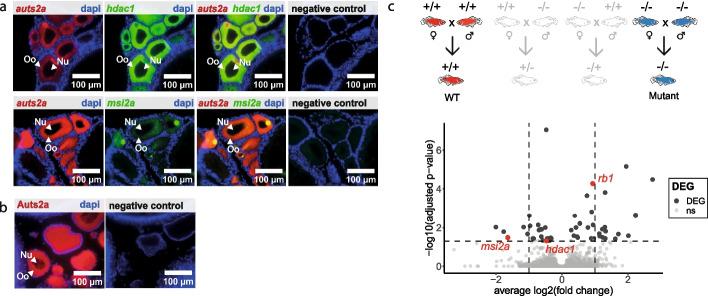
Gene expression in oocytes and one-cell stage RNA-seq analysis.** a** RNAscope (*auts2a* probe and negative control probe) on ovarian sections. RNAscope probe staining is displayed in red (*auts2a*) and green (*hdac1* and *msi2a*) and DAPI staining in blue. Oo: ooplasm, Nu: nucleus.** b** Immunostaining (AUTS2 primary antibody and negative control without primary antibody) on ovarian section. Oo: ooplasm, Nu: nucleus**.** Antibody staining is displayed in red and DAPI staining in blue.** c** Volcano plot of dysregulated genes in 1-cell stage embryos following maternal *auts2a* loss-of-function. Wild-type was used as the control condition. Red dots correspond to *msia2*, *hdac1*, and* rb1*. ns: genes that are not significantly differentially expressed between WT and mutant

Vertebrate oocytes contain maternal factors deposited during oogenesis that support early development prior to zygotic genome activation (ZGA) [50]. The abundance of maternally inherited mRNAs is therefore typically very high during the first stages of embryonic development and subsequently decreases throughout early development during a process called the maternal-to-zygotic transition. In medaka, existing data indicate that *auts2a* mRNA levels remain low and stable during maternal-to-zygotic transition in sharp contrast to the typical profiles of maternally inherited factors contributing to early embryonic development [20]. It seems therefore unlikely, even though it cannot be ruled out, that maternally inherited *auts2a* mRNAs are directly involved in the control of early developmental events described here. In addition, we monitored *auts2a* expression in the medaka ovary and observed a strong signal in the oocyte, at both the mRNA and protein levels (Fig. 5a,b). *AUTS2* is known to regulate gene expression through the PRC1.3/1.5-AUTS2 complex [14–16]. We therefore reasoned that *auts2a* could regulate gene expression in the oocyte, leading to the accumulation of specific maternal factors that could, after being inherited by the zygote, act to regulate gene expression during neurodevelopment. We therefore investigated the abundance of maternally inherited mRNAs (also referred to as maternal mRNAs) in 1-cell stage (i.e., stage 2 [31]) embryos originating from *auts2a* LOF females compared to WT females using RNA-sequencing (Fig. 5c, Additional file 9: Table S6). We identified 46 upregulated genes and 28 downregulated genes in 1-cell stage homozygous mutant embryos compared to WT. Collectively, these transcripts have known or predicted functions related to cellular communication, cell migration, cell proliferation, DNA and RNA binding, cell differentiation, and intracellular trafficking that are consistent with a regulatory role during embryonic development. Among the downregulated maternal mRNAs in 1-cell stage mutant embryos compared to WT, *msi2a* and *hdac1*, that both co-localize with *auts2a* in the oocyte at the mRNA level (Fig. 5a) were of particular interest. *The musashi RNA binding protein 2* (*Msi2*) is a translational regulator that targets genes involved in development and cell cycle regulation. *Msi2* is believed to play a role in the proliferation and maintenance of stem cells, including neuronal progenitor, in the central nervous system [51, 52]. Similarly, we observed a significant depletion in *hdac1* mRNA in 1-cell stage embryos originating from mothers lacking *auts2a* expression. *Hdac1* is an epigenetic regulator known as a transcriptional repressor and acting as a component of the histone deacetylase complex. In all investigated animal species, *Hdac1* is expressed in the oocyte [53]. In mice, Hdac1 regulates cell cycle and zygotic genome activation [53, 54] and is critical for preimplantation development [55]. In zebrafish, *hdac1* plays major role in central and peripheral nervous system development [56]. Among the upregulated maternal mRNAs in 1-cell stage mutant embryos compared to WT, *rb1* is of particular interest. *RB transcriptional corepressor 1* (*rb1*) is involved in heterochromatin formation by maintaining overall chromatin and transcriptional repression by recruiting a histone deacetylase (HDAC) complex for instance [57, 58]. Our observations suggest that maternal *auts2a* directly or indirectly regulates the abundance of maternal factors (including transcriptional regulators) accumulated in the oocyte that are known to regulate early embryonic development and neurodevelopment in the progeny.

### Evolutionary conserved genes linked to behavior and nervous system development

**Fig. 6:**
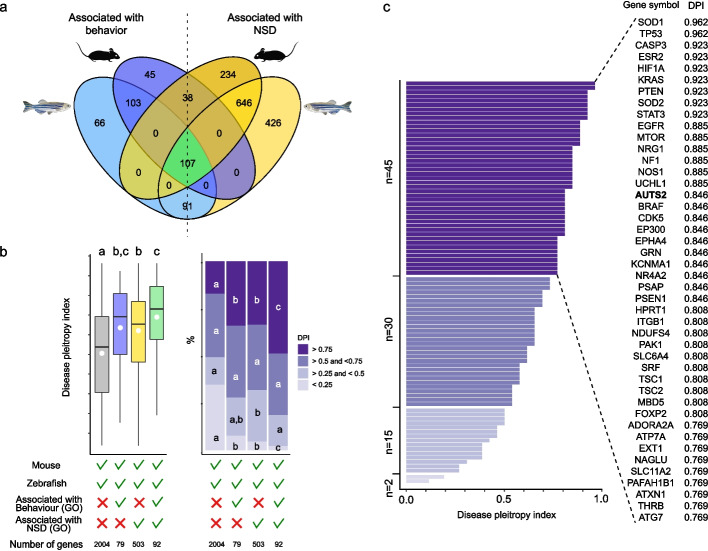
Evolutionarilyconserved oocyte genes linked to behavior and nervous system development.** a** Venn diagram displaying zebrafish and mouse oocyte genes associated with either behavior or nervous system development (NSD). The green area corresponds to genes expressed in oocytes of both species that are associated with both GO terms. **b** Boxplots (left panel) show the distribution of the disease pleiotropy index (DPI) of human gene orthologs to all common zebrafish and mice oocyte genes (grey), genes associated with behavior GO term (blue), NSD GO term (yellow), or both GO terms (green). The number of genes is indicated for each category. For each group of genes, bar plots (right panel) show the proportion of genes in each DPI class. Within each group of genes, differences between DPI classes were assessed using chi square tests. Groups sharing the same letter are not significantly different (*P* < 0.05). **c** DPI of human orthologs of evolutionarily conserved oocyte genes associated with behavior and neurodevelopment (*N* = 92). Symbols of genes with DPI above 0.75 are displayed

The present study is a unique example demonstrating that maternal, but not paternal, LOF of a single gene linked to a human neurodevelopmental syndrome can trigger major neurodevelopmental and long-term behavioral differences in the progeny. We therefore aimed at identifying other genes associated with NDDs that could exhibit similar features. We reasoned that relevant genes had to be expressed in the oocyte [59] and used this criterion to search publicly available gene expression data (see Methods for details). We used the mouse and the zebrafish (another fish model, more commonly used than the medaka), two popular model vertebrate species that are heavily used for biomedical purposes. We identified 11,521 and 16,753 transcripts expressed in mice and zebrafish oocytes, respectively. To subsequently link candidate genes to human pathologies, zebrafish and mouse lists were filtered for common orthologs (Additional file 10: Table S7), which led to the identification of a list of 6205 evolutionarily conserved oocyte-expressed mRNAs. These conserved oocyte genes were subsequently filtered for genes that could be associated with either “Behavior” (GO:0007610) or “Nervous system development (NSD)” (GO:0007399) in either humans, mice (*Mus musculus*), rat (*Rattus norvegicus*), or zebrafish (*Danio rerio*) using the gene ontology database (http://geneontology.org/, see Methods for details). We were able to identify 107 oocyte genes associated with “Behavior” and “NSD” GO terms in both mice and zebrafish (Fig. 6a). We then used the DisGeNET database to identify genes linked to human pathologies. We also used the Disease Pleiotropry Index (DPI) that ranges between 0 and 1 and reflects the diversity of associated diseases; a DPI of 1 corresponding to the highest diversity. Among the 107 identified genes, 92 had a human ortholog present in the DisGeNET database (Additional file 11: Table S8). The DPI of these genes (green box plot, Fig. 6b) was significantly higher than the DPI of genes associated with neither “Behavior” nor “NSD” GO terms (grey box plot, Fig. 6b). Similarly, the DPI of the genes associated with both “Behavior” and “NSD” GO terms (green box plot, Fig. 6b) was significantly higher than the DPI of the genes associated only with “NSD” (yellow box plot, Fig. 6b). We then focused on the genes associated with a higher diversity of diseases (i.e., DPI > 0.75). Among the evolutionarily conserved oocyte genes associated with both “Behavior” and “NSD” GO terms, 49% (45/92) had a DPI above 0.75, including *AUT2* and *EP300* (Fig. 6c), another member of the PRC1.3/1.5-AUTS2 complex [14–16]. The proportion of genes with a DPI above 0.75 was significantly lower for genes associated only with either one of these two GO terms, and for genes not associated with either one of these categories. These results indicate that *AUTS2* and *EP300*, its partner in the PRC1.3/1.5-AUTS2 complex that transcriptionally regulates central nervous system (CNS) genes [14–16], belong to a group of 45 evolutionarily conserved oocyte genes that are associated with “Behavior” and “NSD” and linked to a wide diversity of human pathologies (Fig. 6c). The expression of *Auts2* in the oocyte of both zebrafish and mice revealed by this analysis using publicly available data (see Methods for details) is consistent with the recent report of *AUTS2* expression at significant levels in the human oocyte using RNA-seq [60]. Together with our results, these data suggest that *Auts2* expression in the oocyte is an evolutionarily conserved feature in vertebrates. In addition to *AUTS2 and EP300*, our results also highlight other NDD-associated genes that appear to be expressed in the oocyte in an evolutionarily conserved manner across vertebrates. Among these genes are *PTEN*, *NRG1*, *CDK5*, *GRN*, *EPHA4*, *PAK1*, *MDB5*, and *NR4 A2* that are associated with various NDDs in humans, including schizophrenia [61], Parkinson disease [62], intellectual developmental disorder [63, 64], Alzheimer disease [60, 65], and autism spectrum disorder [64, 66, 67].

## Discussion

*AUTS2* is an important gene for neurodevelopment in vertebrates that has received long-term attention due to its link with a diversity of neurodevelopmental disorder features including intellectual disability, microcephaly, short stature, and developmental delay, known as the AUTS2 syndrome [13, 26]. *AUTS2* acts during development as a transcriptional regulator of CNS genes through the PCR1.3/1.5-AUTS2 complex [15]. Here we report a previously unsuspected non-genetic maternal effect and show that *auts2a* expression in the mother indirectly drives proper nervous system development with major long-term consequences on progeny behavior in medaka. We provide evidence that *auts2a* regulates maternal factors (i.e., maternally inherited mRNAs) in the oocyte that could, in turn, control gene expression during early development, possibly through epigenetic modifications or RNA-binding activity. However, we cannot rule out that other mechanisms including modification of epigenetic marks of the female gamete genome or non-coding RNAs could also contribute to the observed non-genetic effect reported here.

We show that maternal *auts2a* expression has major consequences beyond zygotic genome activation, during neurodevelopment. More specifically, early in neurodevelopment (i.e., late neurula stage), it regulates the abundance of numerous transcription factors as well as genes associated with NDDs, including in neural cells. Maternal *auts2a* expression is also responsible for regulating key neurodevelopmental pathways early in neurodevelopment (i.e., at late neurula stage), and more specifically axon guidance, Wnt signaling, and pathways regulating stem cells pluripotency. Wnt signaling is active in the CNS, orchestrating a broad range of neurodevelopmental processes including CNS regionalization [68]. Further, it represents a mechanistic link among diverse mental illnesses, including ASD [68]. Together, our data show that maternal *auts2a* expression intergenerationally regulates molecular pathways that are required for normal neurodevelopment as illustrated by a significant enrichment in genes associated with NDDs among differentially expressed genes.

The main characteristics of AUTS2 syndrome are global developmental delay or intellectual disability in 97% of patients, microcephaly in 65%, feeding difficulties in 60%, attention deficit hyperactivity disorder or hyperactivity in 62%, autistic traits in 56%, and short stature in 44% [26]. Here we report that several morphological and behavioral features are under maternal *auts2a* control. The reduced head size and total length observed in our model appear to be mostly driven by maternal *auts2a*, rather than by *auts2a* zygotic expression. Similarly, the absence of maternal *auts2a* expression triggered a slower recognition of the environment and a reduced anxiety-like behavior. These results were observed after deleting a significant (90%) portion of the gene sequence corresponding to exons 3–7, including very large intronic sequences, known to be functionally important in mammals [19, 28, 29]. Existing data in humans [12, 26, 27, 30, 69–71] and model species [15, 23, 33] have revealed that AUTS2 syndrome phenotype severity depends on the genomic location of the mutation. In addition to these in vivo studies, yeast two-hybrid screens revealed that Auts2 isoforms have distinct intra-cellular localizations and functions [29]. Our findings suggest that the N-terminal region of the protein encoded by exons 3–7 and/or the genomics regions corresponding to introns 2 to 7 in medaka plays a major role in the maternal determinism of neurodevelopment and behavior reported here. However, it is possible that other parts of the gene, including exons coding for the C-terminal part of the Auts2a protein are important for mediating zygotically expressed *auts2a* functions during development. This hypothesis would be fully consistent with the role of AUTS2 in recruiting P300 in the PRC1.3/1.5-AUTS2 complex through an HX repeat sequence encoded by exon 9 [16], which is located downstream of the region deleted in our study. Further analyses are required to disentangle the role of the different parts of *auts2a* gene in the maternal and zygotic regulation of neurodevelopment and behavior.

We report here a parent-of-origin effect that has never been explored in mammals. Among the patients with pathogenic variants reported to date, most cases involved de novo intragenic deletions, precluding the investigation of parent-of-origin effects. In addition, the wide range of *AUTS2* mutations complicates comparisons based on parental origin. Indeed, it cannot be excluded that only specific parts of the gene (possibly the N-terminal part based on our data) are involved in the non-genetic inheritance effect reported here. Furthermore, existing studies in mice were not experimentally designed to study and identify a parent-of-origin effect. Because a genome-wide *Auts2* full knock-out is embryonically lethal, most studies [15, 32, 72–75] performed conditional knock-outs in the brain that excluded the possibility to investigate the consequence of the lack of *Auts2* expression in the oocyte and reveal any subsequent maternal effect. In another study [23], the experimental design was different but the studied heterozygous progeny automatically inherited the mutant *Auts2* allele from their father, making it impossible to observe a parent-of-origin effect. Even though our results do not contradict existing studies in mammals, the existence of the maternal non-genetic effect reported here in fish remains to be investigated in other vertebrate clades.

## Conclusions

We describe here the unexpected role of an NDD-associated gene in maternally regulating progeny neurodevelopment and behavior. This model constitutes a unique example of a non-genetic inheritance mechanism controlling progeny neurodevelopment and behavior. We also identified a list of 45 vertebrate genes sharing similar features including oocyte expression, association with NSD and behavior as well as a link with a large number of human diseases. Our data suggest that evolutionarily conserved mechanisms and genes intergenerationally regulate neurodevelopment and behavior in vertebrates. This novel finding paves the way for studying intergenerational effects on behavior in various other contexts, including in response to maternal stress. Because of the observed association with multiple diseases, it also raises the question of the intergenerational determinism of NDDs, which has received no or very little attention thus far, including in the case of the AUTS2 syndrome.

## Methods

### Fish rearing

Adult medaka (*Oryzias latipes*) from the HdrR strain were raised at 26 °C under 12 h light/12 h dark photoperiod for 3 months then transferred to a long-day photoperiod (15.75 h light/8.25 h dark) to trigger reproduction. The fertilization date and the density of fish (0.3–0.4 L/fish) were similar for fish raised in different tanks. All tanks were part of the same recirculated water system in which physico-chemical parameters and pH of the water were controlled. Fish were fed 4 times daily.

### Creation of mutant lines

Genome editing was performed using CRISPR/Cas9. Identification of target sites was performed using the CRISPOR tool (http://crispor.org) [76]. The following guide sequences were used: GCTCGAGTGGTGTCACAGTTTGG (Exon 2, 5′ UTR), GTCTAACCTGGACACAGGCTTGG (Intron 2), GATCAAAGTATCAGTCAGAAAGG (Intron 6), and TCTCACATCATCTCATGTTCTGG (Intro 7). Guide RNA were prepared as previously described [77] using the DR274 plasmid (Addgene plasmid # 42,250). For the Cas9 guide RNA, the pCS2-nCas9n plasmid (Addgene plasmid #47,929) was linearized using Not1 before RNA synthesis. One-cell medaka embryos were injected with 50 ng/µl of each guide RNA, 150 ng/µl of Cas9 RNA, and 200 ng/µl Cas 9 protein (Invitrogen, Waltham, MA, USA). Injected individuals were screened (see genotyping procedure below) 2 months after injection and fish harboring a suitable deletion of the targeted zone were crossed with wild-type individuals to generate a stable line. The mutant line was maintained over generations using mixed-sex heterozygous individuals. When necessary, these heterozygous fish were crossed to generate homozygous mutants (−/−) and WT for the experiments. Thus, mutant and WT used for each crossing originate from the same parents that have been raised together in common tanks until genotyping (at adult stage).

### Genotyping

Adult fish used in all experiments were anesthetized and a small piece of the caudal fin was biopsied for genomic DNA extraction under anesthesia. Fin samples were lysed in 35 µL of lysis buffer containing 1.25 M NaOH and 10 mM EDTA (pH 12) incubated at 95 °C for 45 min and the lysis reaction was neutralized by adding 35 µL of neutralization solution containing 2 M Tris–HCl (pH 5). The identification of wild-type and mutant fish was performed by PCR using primers flanking the site of deletion. PCR was performed using the Jump Start polymerase and the following PCR conditions: 94 °C for 2 min, followed by 37 cycles (94 °C/30 s, 60 °C/30 s, 72°/2 min) and a final 2 min step at 72 °C. The following primers were used: Auts2 gN_extF (CTTGAAGAACGCTGCTGTTG), Auts2 gN_extR (CTGCACAAACAGCTCTGCAT), Auts2 gN_intF (AAACCCACAGCCAACATTTC), and Auts2 gN_intR (GGCAGTGCCACACTGTTTAG). The efficiency of the genomic deletion was verified using RNA-seq data (see below). No reads mapping to the Δ338 K genomic deletion could be detected in pools of homozygous embryos at the three investigated stages.

### Macroscopical phenotyping

For embryo survival, fertilized eggs were collected directly from the female approximately 10 min after fertilization. Embryonic survival was monitored at stage 18 (i.e., late neurula, 25 hpf), stage 20 (i.e., 4-somite, 1 d 7.5 hpf), stage 25 (i.e., 18-somite, 2 d 2 hpf), stage 29 (i.e., 34-somite, 3 d 2 hpf), stage 32 (i.e., somite completion, 4 d 5 hpf), and stage 29 (i.e., hatching, 8/9 dpf). Dead embryos were counted and removed.

For head size and body length measurement, anesthetized fish were placed in a lateral position on a Petri dish with a ruler, and photographed. The images were then imported into Fiji software, and a 1-cm distance was converted into pixels for each picture. For the head measurement, a horizontal line was drawn from the anterior edge of the skull to the posterior margin of the head. The same technique was used to measure total length, from the anterior edge of the skull to the tip of the caudal fin. Each measurement was performed in triplicate, and the average was calculated to obtain robust values.

No effect of the mutation on the sex ratio was observed neither in heterozygous (+/−) groups nor in homozygous groups (WT and mutant).

### Behavioral phenotyping

The experimental protocol for novel tank and recognition tests is depicted in Fig. 2a. Sixty-two 14-week-old naïve fish were tested. Each fish came from stock populations and was kept alone in a 3-L aquarium (Home tank) for 3 days before the beginning of the test. The temperature of the home tank water was maintained at 26 ± 0.5 °C. One fish adapted to its home tank was carefully transferred to a 3.4-L cubic test tank at 9 a.m. Video was recorded for 1 h and fish were kept in their test tank until 5 p.m. (habituation period). They were then carefully transferred to their original home tank for the night. On day 2, the fish was transferred back into the test tank at 9 a.m. and video recorded for 1 h. Then, fish were euthanized and a small piece of caudal fin was sampled for genotyping. No significant difference in sex ratio was observed in any of the experimental groups. During video recording, fish were alone in the room to avoid human-induced bias. Fish activities in the test tank were analyzed using the EthovisionXT software (v. 15.0. 1418) (Noldus, Wageningen, The Netherlands).

The experimental protocol for larval behavioral phenotyping was adapted from a previously published procedure on 5 dpf zebrafish larvae [35] and is depicted in Additional file 2: Fig. S2a. A total of 113 4-day-old larvae, which had hatched at 9 days post-fertilization, were tested. The experiment was conducted using 12-well plates in an automated video recording system (Zebrabox Imaging System) with one larva placed in each well. Following a 30-min habituation period under 300-lx light, the larvae were subjected to a stressing dark challenge phase lasting 10 min. During these 10 min, the time spent in the inner and outer zones of the plate was measured; the time spent in the outer zone being used as an indicator of anxiety-like behavior.

### RNA-sequencing

#### Sample collection

Fertilized medaka eggs were collected, and developing embryos were subsequently incubated at 27 °C in mineral medium (water, 0.1% NaCl, 0.003% KCl, 0.004% CaCl_2_, 0.016%, MgSO4) until they reached the targeted developmental stage (stage 2, 18, or 29) according to Iwamatsu [31]. Stage 2 corresponds to the 1-cell stage [31]. At that stage all detected mRNAs are of maternal origin [50]. Stage 18 corresponds to the late neurula stage (early stage of neurodevelopment) [31] and stage 29 to the late embryonic brain formation (late stage of neurodevelopment) [31]. Both stages 18 and 29 occur after zygotic genome activation. In teleost fishes, zygotic genome activation starts around 64-cell stage (stage 8 in medaka) [78]. Most of the zygotic genes are activated before the mid-blastula transition (stage 10 in medaka) [79, 80]. Embryos were then snap frozen in liquid nitrogen and stored at − 80 °C until RNA extraction. For stage 2 analysis, mutant and WT group embryos were collected and 5 biological replicates, corresponding to pools of 100 embryos originating from 5 different female were used for each group. For stage 18 and 29 analyses, HME − and HME + embryos were collected and 6 biological replicates, corresponding to pools of 50 embryos originating from 6–10 females, were used for each group.

#### RNA extraction

Frozen medaka embryos were lysed with a Precellys Evolution Homogenizer (Ozyme, Bertin Technologies, Montigny-le-Bretonneux, France) in TRI Reagent (TR118; Euromedex, Cincinnati, OH, USA), and total RNA was extracted according to the manufacturer’s instructions. Due to the large amount of yolk at stage 2 (one-cell stage), these samples were additionally purified using NucleoSpin RNA plus kit (Macherey–Nagel, Düren, Germany). After extraction, the RNA concentration was measured using a Nanodrop ND-1000 (Nixor Biotech, Paris, France) and sample integrity was checked using a 2100 Bioanalyser RNA 6000 nano kit (Agilent technologies, Santa Clara, CA, USA) and/or Fragment Analyzer RNA Standard Sensitivity kit (Agilent technologies, Santa Clara, CA, USA). RNA was stored at − 80 °C until library synthesis.

#### Library synthesis

All libraries were synthetized from at least 2 µg of template RNA. For stage 18 and 29 embryos, polyadenylated RNAs were captured using oligo(dT) magnetic bead and libraries were synthesized using Stranded mRNA Prep Ligation (Illumina, San Diego, CA, USA). For stage 2 embryos, as maternal mRNAs have little or no polyadenylation, ribosomal RNA was depleted using riboPOOLs kit (siTOOLs Biotech, Rückgebäude, Germany) and library synthesis was performed on remaining RNAs using TruSeq Stranded mRNA Sample Preparation kit (Illumina, San Diego, CA, USA). Briefly, RNAs were chemically fragmented and synthesis of the first strand of cDNA was made in presence of actinomycin D to block second strain synthesis. The second strain of cDNA was synthesized with dUTP to block its synthesis during PCR amplification. Adapters were ligated to the 5′ end (P5) and 3′ end (P7) and cDNAs were then amplified by PCR. PCR products were purified using AMPure XP Beads (Beckman Coulter Genomics, Brea, CA, USA). Libraries were quantified and their quality checked using the Fragment Analyzer NGS High Sensitivity kit (Agilent technologies, Santa Clara, CA, USA) and KAPA Library quantification kit (Roche, Basel, Switzerland).

#### Clustering and sequencing

Clustering and sequencing were performed on a NovaSeq6000 (Illumina, San Diego, CA, USA) using NovaSeq Reagent Kits (Illumina, San Diego, CA, USA) and the SBS (Sequence By Synthesis) technique. To avoid any bias in HME analysis, stage 18 and 29 HME + and HME − samples were processed on a single-read 100 bp flow cell SP and stage 2 WT and mutant samples on a second flow cell. More than 1073 million reads were obtained with a number of reads per library ranging from 32 to 60 million for stage 18 and 29 HME +/HME − samples. More than 779 million reads were obtained with the number of reads per library ranging from 60 to 105 million for stage 2 samples.

#### RNAseq analysis

Raw read adapter and quality trimming were performed using Trim Galore (version 0.6.6, Cambridge, UK) [81] (parameters: –clip_r1 13, –three_prime_clip_r1 2). Recent publications highlighted improvement in transcriptome alignment and quantification accuracy using Salmon through Selective Alignment mode [82, 83]. Trimmed reads were therefore mapped against *Oryzias latipes* (ASM223467v1 Ensembl version 106) coding sequence as transcriptome and genome as decoy sequences, and subsequently quantified using Salmon (version 1.8) through Selective Alignment mode based. These steps have been gathered in the form of a RNAseq workflow in accordance to FAIR principles (https://forgemia.inra.fr/lpgp/rnaseq). Genes (pseudo) counts (≥ 40 reads per gene in at least 4 of the 5 WT samples for stage 2 analysis and in at least 5 of 6 samples per condition for stage 18 and 29 analysis), normalization and differential gene expression analysis have been carried out using DESeq2 package (version 1.38.2) on R software (version 4.2.2) [84] using default parameters.

### Single-nuclei RNA-sequencing

#### Sample collection

Fertilized medaka eggs were collected and incubated at 27 °C in mineral medium (water, 0.1% NaCl, 0.003% KCl, 0.004% CaCl_2_, 0.016% MgSO_4_) until 2–3 h before reaching the targeted embryonic developmental stage (stage 18) according to Iwamatsu [31]. Embryos were then incubated in protease (20 mg/mL—P8811, Sigma-Aldrich, Saint-Louis MO, USA) for 1 h, washed twice in balanced salt solution (BSS: water, 0.65% NaCl, 0.04% KCl, 0.02% MgSO_4_, 0.02% CaCl_2_, 0.01% NaOHCO3, 0.00005% Phenol Red), incubated in medaka hatching enzyme for 1–2 h and washed in BSS. Chorions were removed and embryos were deyolked using thin forceps, subsequently snap frozen in liquid nitrogen and stored at − 80 °C until nuclei dissociation. Pools of 50 embryos originating from several parents were collected for HME + and HME −.

#### Nuclei dissociation

Pools of embryos were resuspended in 1 mL of Ice-cold Nuclei Extraction Buffer (Miltenyi Biotec, Bergisch Gladbach, North Rhine-Westphalia, Germany) with RNAse inhibitor (40U/µL – Promega, Madison, WI, USA) and RNAse Protector (40U/µL – Roche, Basel, Switzerland), dissociated and incubated on ice for 5 min. The dissociation solution was filtered through a 70-µm MACS SmartStrainer (Miltenyi Biotec, Bergisch Gladbach, North Rhine-Westphalia, Germany). Samples were centrifuged 5 min (4 °C—500* g*), and nuclei pellets were resuspended in 1 mL of PBS 1X—BSA 1% with RNAse inhibitor (40U/µL—Promega, Madison, WI, USA) and RNAse Protector (40U/µL—Roche, Basel, Switzerland). Samples were then filtered through a 30-µm MACS SmartStrainer (Miltenyi Biotec, Bergisch Gladbach, North Rhine-Westphalia, Germany).

#### Fluorescence-activated cell sorting

The morphology of nuclei was checked using a microscope (Nikon Eclipse 90i epifluorescence microscope) and Hoechst staining. Nuclei were then sorted using a BD FACS Aria Fusion sorter. A total of 50,000 nuclei were collected per replicate in ice-cold PBS 1X/BSA 1%.

#### Single-nuclei RNA sequencing

Nuclei pre-mRNAs were sequenced using the Chromium Next GEM Single Cell 3′ Reagent Kit v3.1 (10X Genomics) following the manufacturer’s instructions. Twenty thousand nuclei were loaded to the 10X Genomics Chromium Next GEM Chip G (10X Genomics). Library quality was checked using an Agilent Bioanalyzer High Sensitivity (Agilent technologies, Santa Clara, CA, USA). Libraries were sequenced independently using the Novaseq 6000 platform (Illumina, San Diego, CA, USA) to reach 58,000–61,000 reads per cell. Cell Ranger version 6.1.1 (10X Genomics) was used to align reads on the medaka reference genome (ASM223467v1 Ensembl version 106) and to produce the count matrix. CellBender algorithm (version 0.2.2) was used to remove ambient RNA.

#### snRNA-seq analysis

All statistical analyses were performed using R software (v4.3.1). snRNAseq data analysis was performed using the R packages Seurat (version 5.0.1) and SeuratObject (version 5.0.1) and are publicly available (https://github.com/cmerdrignac/auts2a).

#### Quality control and filtering

Five thousand nine hundred thirty eight droplets (HME + dataset) and 6054 droplets (HME − dataset) were filtered on the basis of three standard quality control criteria: the number of unique genes detected, the total number of molecules detected, and the percentage of reads that map to the mitochondrial genome. Droplets with less than 500 genes, less than 1000 molecules, or that had more than 5% of their reads from mitochondrial genes were removed. Gene expression was normalized using the NormalizeData() function, the 2000 most variable genes were identified using the FindVariableFeatures() function. The normalized expression of these top genes was scaled and standardized across all nuclei (*z*-score transformation) with the three quality control variables mentioned above using ScaleData() function, and used for dimensional reduction with principal components analysis (PCA) at 50 dimensions using RunPCA() function. Clustering was then performed using Find Neighbors() and FindClusters() functions (30 PCs; resolution = 0.5). Clusters were visualized in two dimensions using the RunUMAP() function (dims = 1:30; minimum distance = 0.3; n_neighbors = 30 L; metric = ‘cosine’). We then identified and removed droplet doublets using DoubletFinder R package (version 2.0.3).

#### Integration and clustering analysis

Features that were repeatedly variable across the HME + and HME − batches were selected to perform integration using the IntegrateData() function. Integrated data were scaled based on the regression of the three quality control variables mentioned above using ScaleData() function, and used for dimensional reduction with principal components analysis (PCA) at 50 dimensions using RunPCA() function. Clustering was then performed using FindNeighbors() and FindClusters() functions (30 PCs; resolution = 0.5). Clusters were visualized in two dimensions using the RunUMAP() function (dims = 1:30; minimum distance = 0.3; n_neighbors = 30 L; metric = ‘cosine’). We then performed differential expression analysis and annotated clusters as detailed below. FindAllMarkers() function was used to identify the top 20 gene markers for each cluster (Additional file 5: Fig. S3a, Additional file 6: Table S3). Their expression was subsequently investigated using the ZFIN database [85] at the corresponding developmental stage (10 hpf) and the previously annotated dataset of single cell zebrafish embryo at the corresponding developmental stage (10 hpf) [86]. We observed a small cluster (undefined) formed by low-quality cells, expressing high levels of ribosomal proteins. This cluster was not taken into account in the analysis.

#### Differential expression analysis

To identify differentially expressed genes within a unique cell population between HME + and HME −, we performed a nonparametric Wilcoxon rank sum test using the FindMarkers() function with default parameters.

#### KEGG pathway enrichment analysis

Human ortholog of differentially expressed genes were found using getBM() function from the biomaRt package (version 2.58.0). Enrichment analysis was performed on these human orthologs using enrichKEGG() function from the KEGGREST package (version 1.42.0). Covid19 disease was deliberately excluded from the enriched KEGG pathways due to its overrepresentation in recent studies.

#### Transcription factor analysis

Transcription factors were identified by crossing human orthologs (identified with biomaRt as previously described) of differentially expressed genes from RNA-seq analysis with the human transcription factor catalog [36].

#### Association with human diseases

Human-medaka gene orthology relationships were assessed using the Genomicus web server (https://www.genomicus.bio.ens.psl.eu/genomicus-fish-03.01/cgi-bin/search.pl). The complete list of human-medaka orthologs sharing the same Euteleostomi gene ancestor can be downloaded via an ftp server (ftp://ftp.biologie.ens.fr/pub/dyogen/genomicus-fish/03.01/ancGenes.tgz). Genes associated with human diseases were subsequently obtained using the DisGeNET database (https://www.disgenet.org/search). This database integrates information on human gene-disease associations from various repositories including Mendelian, complex, and environmental disease [37]. Our gene datasets were compared to a random selection in the Japanese medaka genome. The process of random selection in the Japanese medaka genome involves averaging ten random draws from a pool of referenced coding genes (https://www.ensembl.org/biomart/martview/54025bd283efc6f4758f0a040f518884). The number of genes selected randomly matches the number of conditions being compared (i.e., 401 genes for DEG, 196 for neural DEG and 139 for exclusive neural DEG).

### RNAscope

RNAscope is an in situ hybridization method which allows the spatial detection and quantification of mRNAs using a commercially designed probe. The *auts2a* probe and the negative control probe used were the same as described in our previous study [20]. The RNAscope *hdac1* and *msi2a* probes were designed by ACDBio company. The *hdac1* probe consisted of 19 ZZ probes targeting nucleotides 2–2218 of the Japanese medaka *hdac1* full-length transcript (XM_011490522.3). The *msi2a* probe consisted of 20 ZZ probes targeting nucleotides 151–1326 of the Japanese medaka *msi2a* full-length transcript (XM_023962327.1). Ovary sections were obtained and sample pretreatment and unmasking steps were performed as previously described [77]. RNAscope was carried out using the RNAscope Multiplex Fluorescent V2 kit (ACDBio, ref: 323,100) following the manufacturer’s instructions (ACDBio, bio-techne, Newark, CA, USA).

### Immunofluorescence

#### Ovary sampling and section preparation

Tissue preparation protocol was established based on previous studies and technical resources [87, 88]. Female medaka were euthanized by an overdose of tricaine at 300 mg/L (tricaine, PharmaQ, Overhalla, Norway) diluted in water with 600 mg/mL of sodium bicarbonate (S5761, Sigma-Aldrich, Saint-Louis MO, USA). After removing the head and tail, the body cavity was opened to eliminate non-reproductive organs, and ovaries were fixed in situ to maintain their structure. The trunk was briefly rinsed in phosphate-buffered saline (PBS: 137 mM NaCl, 2.7 mM KCl, 10 mM Na2HPO4, 1.8 mM KH2PO4, pH 7) 1X before fixation in 4% paraformaldehyde (PFA)/PBS for 5 h at room temperature (RT) under gentle agitation. Following fixation, ovaries were dissected, briefly rinsed in PBS 1X at RT, and then equilibrated in sucrose diluted in PBS (25% and 35%) and in OCT (1:1 sucrose 35% diluted in PBS 1X/OCT (M-1 embedding matrix, Thermo Fisher Scientific, Waltham, MA, USA)) 2 h at RT under gentle agitation for each step. Subsequently, ovaries were further equilibrated in 100% OCT for 30 min at RT before being frozen in cold isopentane for 1 min and stored at − 80 °C. Ovary sections of 20-μm thickness were obtained using a Cryostat (3050S, Leica Microsystems, Wetzlar, Germany), placed on adhesive glass slides (Superfrost Plus, VWR, Radnor, PA, USA) and dried for 2 h at RT.

#### Immunostaining

Sections were first incubated in a blocking solution (Tris-Buffered Saline (TBS), 10% Normal Goat Serum (NGS), 0.025% Triton X-100, 1% Dimethylsulfoxyde (DMSO)) for 2 h at RT. AUTS2 primary antibody (SAB2700423, Sigma-Aldrich, Saint-Louis MO, USA) diluted (1/200) in an antibody solution (TBS, 10% NGS, 0.025 Triton X-100) was applied overnight at 4 °C. Sections were then washed twice in a washing buffer (TBS, 1% DMSO) for 5 min each at RT. Negative control sections received only the antibody solution without the primary antibody. Secondary antibody (Alexa Fluor 488 goat anti-rabbit, Invitrogen, MA, USA) diluted (1/400) in the antibody solution was applied overnight at 4 °C in the dark. Sections were then washed three times for 5 min each in TBS. Nuclei were counter-labeled with DAPI diluted at 1/100 in TBS, for 30 s at RT in the dark. Cover slides were mounted using ProLong Gold antifade reagent (P36934, Thermo Fisher Scientific, Waltham, MA, USA), and slides were left overnight in the dark to polymerize. Pictures were acquired using a fluorescent microscope (DM6B, Leica Microsystems, Wetzlar, Germany) and processed under Fiji software (ImageJ, release 2.9.0, National Institutes of Health, Bethesda, MD, USA) to adjust the color balance.

### Evolutionarily conserved NDD genes

#### Gene annotation and ortholog identification

Zebrafish-human, zebrafish-mouse, and zebrafish-rat orthology relationships were retrieved from Ensembl using BioMart [63].

#### Gene ontology

Gene ontology was characterized using http://geneontology.org/[89, 90]. The identifiers GO:0007610 and GO:0007399 correspond to “Behavior” and “Nervous system development” terms respectively. Genes of *Homo sapiens*, *Mus musculus*, and *Rattus norvegicus* associated with both GO terms were uploaded.

#### Evolutionarily conserved oocyte genes associated with behavior and neurodevelopment in vertebrates and linked to a diversity of human diseases

Maternal transcriptome datasets from zebrafish (*Danio rerio*) and mouse (*Mus musculus*) were downloaded from NCBI SRA (zebrafish: #SRX3542388, BioProject #PRJNA429172; mouse accession #ERX1787721-32, BioProject #PRJEB17626). The minimum TPM threshold was fixed at 0.5. Lists of genes belonging to “Behavior” and “Nervous system development” biological processes were downloaded (04/2020) from Gene Ontology/Amigo database for 3 mammalian species: rat (*Rattus norvegicus*), human (*Homo sapiens*), and mouse (*Mus musculus*). These species were selected because they were the most described for these gene ontology (GO). All orthologous genes were obtained from BioMart on ENSEMBL platform [63]. GO lists and maternal transcriptomes were merged using Rstudio (R version 3.1.5). The curated gene-disease association lists were downloaded from the DisGeNET website. They contain gene-disease associations from UNIPROT, CGI, ClinGen, Genomics England, CTD, PsyGeNET, and Orphanet. Data were merged using the R software (v4.1.0) with others data frame by “human gene name” [37].

## Statistical analysis

Statistical analyses were performed using R software (v4.1.0)/R commander package. Effects were considered statistically significant at *P* < 0.05. Analyses were carried out using untransformed data when criteria for normality and homogeneity of variances were fulfilled (Shapiro–Wilk and Levene’s test, respectively).

## Supplementary Information

Additional file 1. Fig S1 Mutant auts2a 338 k line information. Fig S2 Behavioral analysis at larval stage. Fig S3 Single nucleus RNA-seq additional information.

Additional file 2. Table S1 List of expressed genes at stage 18 with p values, log2FC and normalized counts (HME RNA-seq analysis). Table S2 List of expressed genes at stage 29 with p values, log2FC and normalized counts (HME RNA-seq analysis). Table S3 List of the top 20 cluster-specific marker genes, with zebrafish ortholog ZFIN expression and the assigned cell type in a previously annotated dataset of single cell zebrafish embryo at the corresponding developmental stage (10hpf). Table S4 List of expressed genes at stage 18 with p values, log2FC and normalized counts (HME snRNAseq analysis). Table S5 List of DEG human orthologs associated with human pathologies, enriched in KEGG pathways and belonging to different categories of transcription factors. Table S6 List of expressed genes at 1-cell stage with p values, log2FC and normalized counts. Table S7 List of maternally conserved genes with associated GO terms. Table S8 List of human orthologs genes associated with diseases and DPI score.

## Data Availability

All transcriptomics datasets generated as part of the study are publicly available. Bulk RNA-seq and single nuclei RNA-seq dataset are available on the NCBI SRA portal under accession numbers PRJNA929688 (bulk RNA-seq; stages 18 & 29) [91], PRJNA930338 (bulk RNA-seq; stage 2) [92] and PRJNA1076130 (snRNA-seq) [93]. Source images are available in Figshare [94]. All codes used for the computational analysis are publicly available as detailed in the methods section. Source code for snRNA-seq [95] and bulk RNA-seq [96] are available in Zenodo (CC BY 4.0).
