# Supplementary material for: Parent-of-origin regulation by maternal auts2 shapes neurodevelopment and behavior in fish
Source: Genome Biol. 2025 May 9;26:125. doi: 10.1186/s13059-025-03600-y (PMC12063280; doi:10.1186/s13059-025-03600-y)
Supplement: Supplementary file 1 — Additional file 1. Fig S1 Mutant auts2a 338 k line information. Fig S2 Behavioral analysis at larval stage. Fig S3 Single nucleus RNA-seq additional information. [file 13059_2025_3600_MOESM1_ESM.pdf]

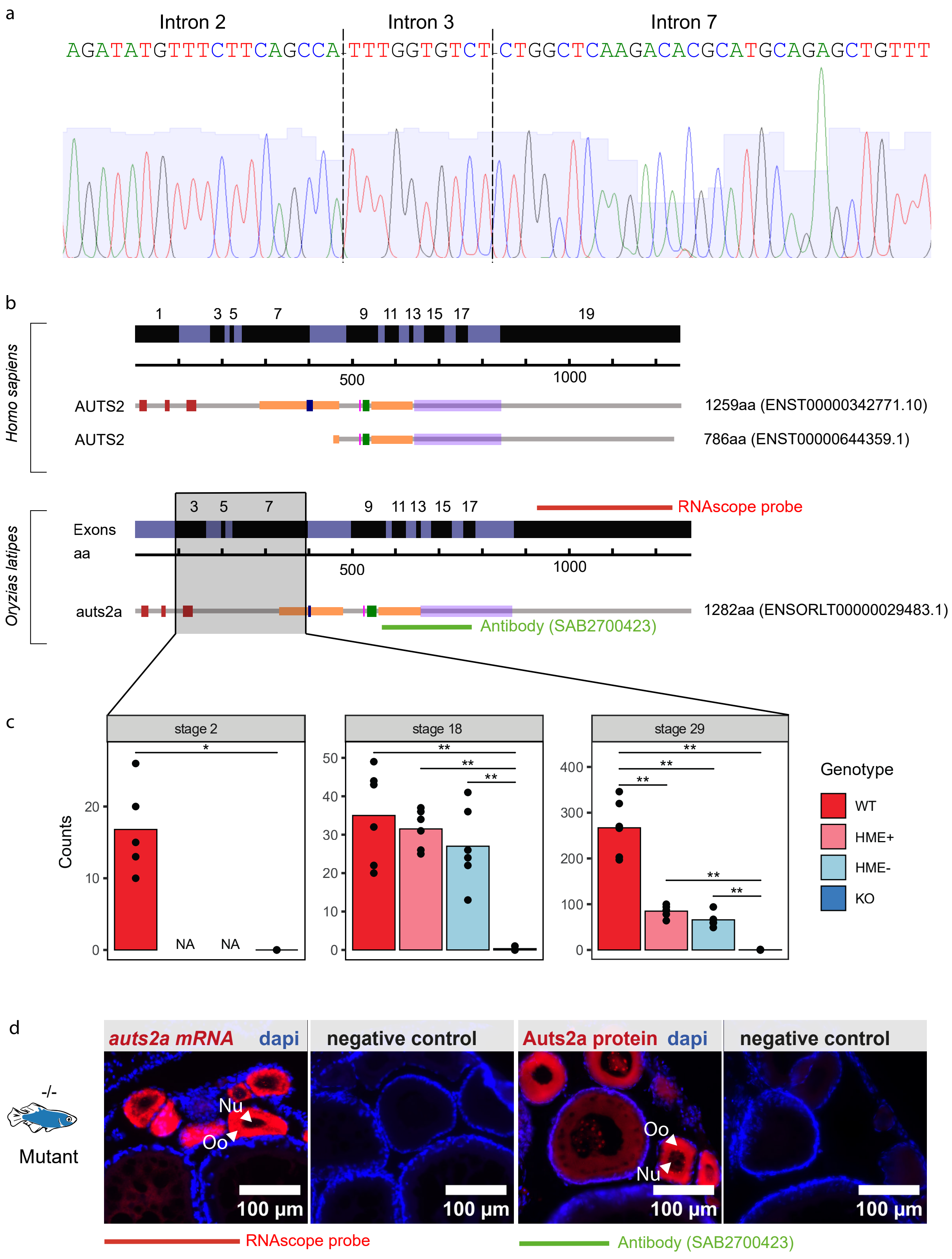

Fig S1. **a** Chromatogram of the mutated genomic region of the *auts2a* 338k line. **b** Scaled diagram of the full and short-length WT and mutant predicted transcripts and proteins in human or medaka. Auts2a protein domains are represented by different colors. Nuclear Localization Signal (NLS, red), Serine Rich Repeat (SRR, blue), Proline Rich region 1 and 2 (PR1 & PR2, orange), PY protein binding motif (PY, pink), Histidine repeats (HX, green), Auts2 family

domain (auts2, purple). **c** Reads mapping in the 338kb deletion (spanning from exon 3 to 7) of the *auts2a* gene at stage 2 (fertilized egg), stage 18 (late neurula) and stage 29 (late embryonic brain formation) for the four groups. Pairwise Wilcoxon tests incorporating the False Discovery Rate correction method were conducted to compare reads counts between genotypes within specific stage and exonic region. NA: not assessed. * P<0.05, ** P<0.01, *** P<0.001. **d** RNAscope and immunostaining on mutant ovarian sections. RNAscope probe staining is displayed in red (auts2a and negative control probe) and DAPI staining in blue. Antibody staining (AUTS2 primary antibody and negative control without primary antibody) is displayed in red and DAPI staining in blue. Oo: ooplasm, Nu: nucleus.

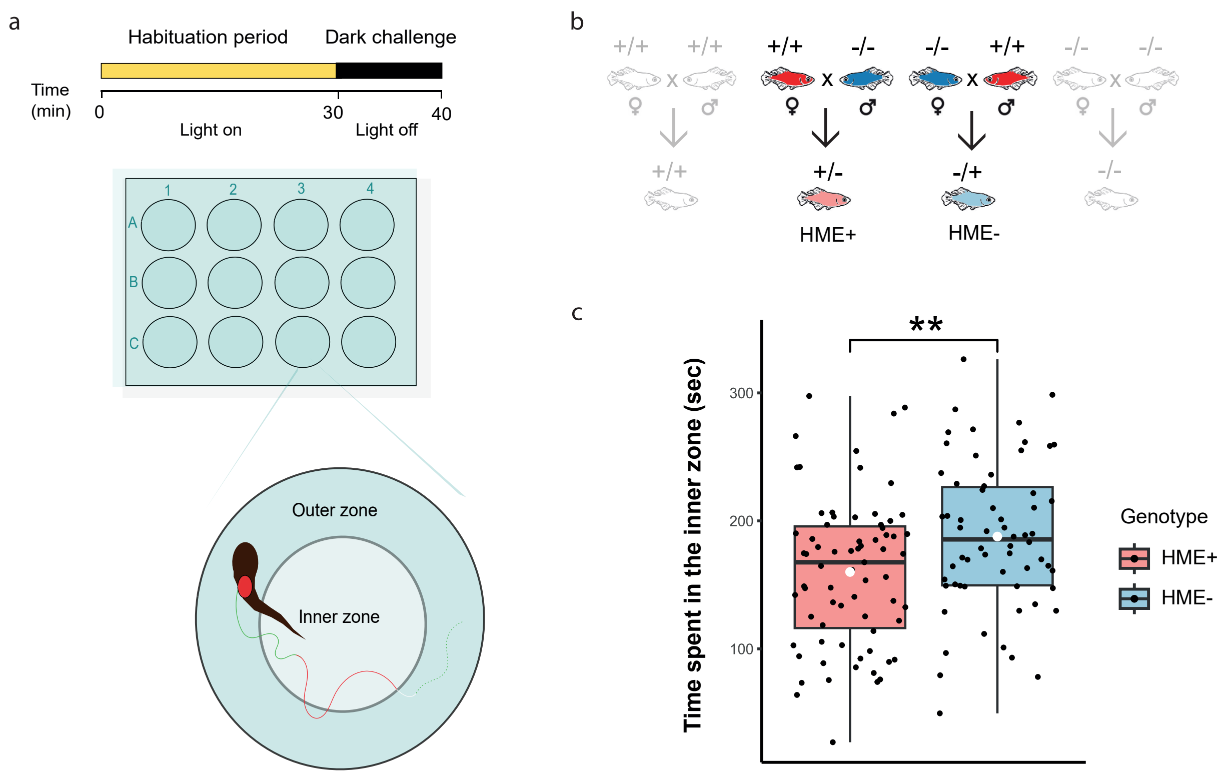

Fig S2. a Schematic representation of behavioral test. **b** Crossing of individuals used for behavioral test to assess the impact of maternal *auts2a* expression. **c** Time spent in the inner zone of the well (in seconds). Student statistical test was performed on HME+ (n=71) and HME- (n=62) groups. ** P<0.01.

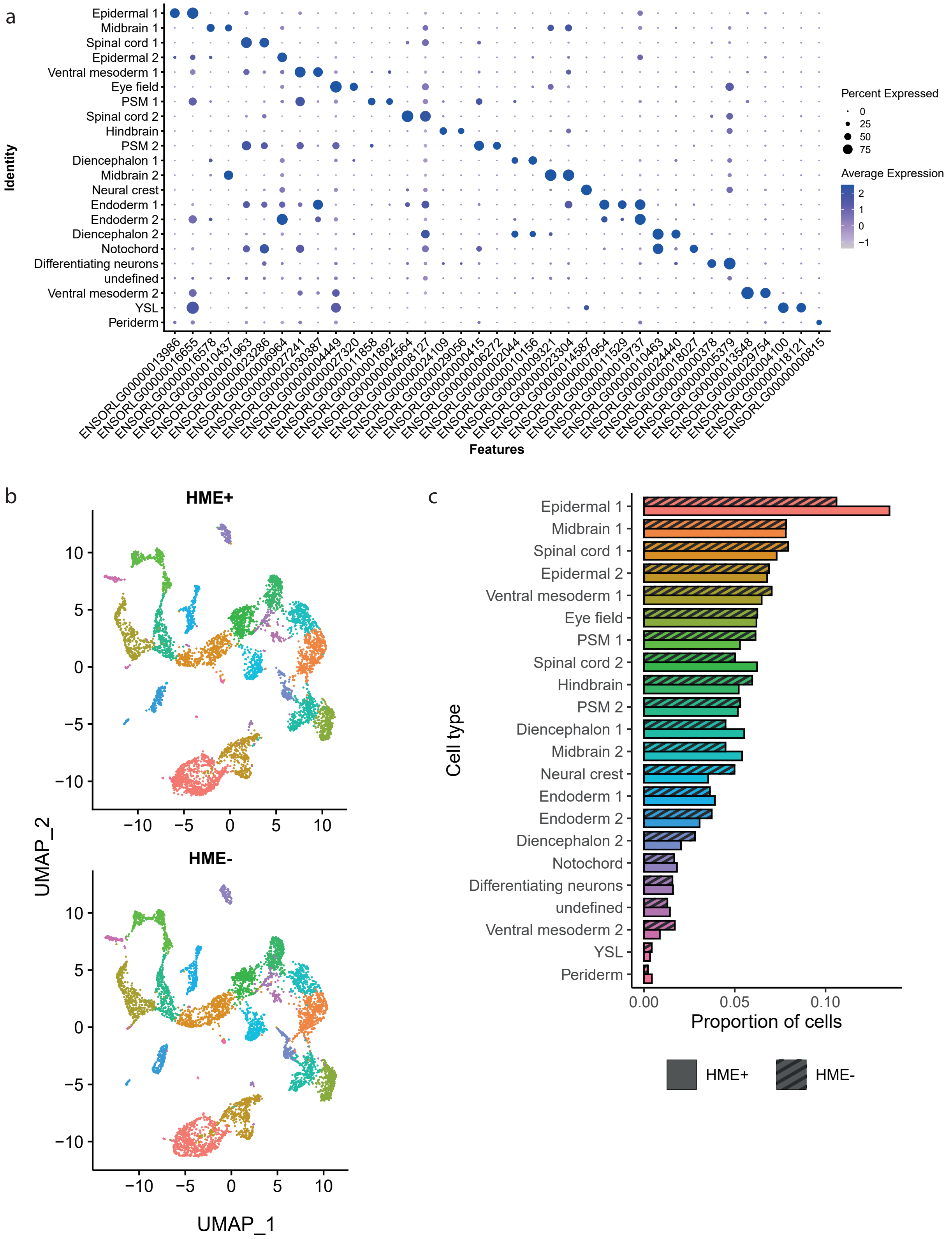

Fig S3. **a** Dotplot display the cellular expression of gene used to assess cell type (see Table S3). **b** snRNA-seq clustering during early stage of neurodevelopment. UMAP constructed in dimensionality-reduced PCA defined by highly variable genes from integrated stage 18 HME+ and HME- datasets (see methods). Cells from stage 18 HME+ datasets are separated from cells from HME- datasets. Cells sharing a same color have the same cell type identity determined from expressed marker genes. **c** Barplot displays the proportion of each cell type for stage 18 HME+ and stage 18 HME- datasets.
